# Calsyntenin 3β Is Dynamically Regulated by Temperature in Murine Brown Adipose and Marks Human Multilocular Fat

**DOI:** 10.3389/fendo.2020.579785

**Published:** 2020-09-25

**Authors:** Kaja Plucińska, Naja Z. Jespersen, Erin L. Brown, Patricia S. Petersen, Kaja Rupar, Søren Nielsen, Camilla Scheele, Brice Emanuelli

**Affiliations:** ^1^Novo Nordisk Foundation Center for Basic Metabolic Research, Faculty of Health and Medical Sciences, University of Copenhagen, Copenhagen, Denmark; ^2^Centre of Inflammation and Metabolism and Centre for Physical Activity Research, University of Copenhagen, Rigshospitalet, Copenhagen, Denmark

**Keywords:** brown adipose tissue, calsyntenin 3-beta, S100B, sympathetic innervation, uncoupling protein 1, UCP1

## Abstract

Activation of thermogenic adipose tissue is linked to improved metabolic outcomes in mice and humans. Dissipation of energy as heat during thermogenesis relies on sufficient innervation of fat by sympathetic nerve fibers, a process recently proposed to be regulated by the adipose-specific calsyntenin3β (Clstn3β)-S100b axis. Here we aimed 1) to assess enrichment patterns of *CLSTN3β*, *S100b* as well as the previously annotated neuronal *CLSTN3α* in perirenal brown and subcutaneous white human fat specimens, and 2) to investigate if the novel *Clstn3β* is dynamically regulated by changes in environmental temperatures and nutritional stress in thermogenic adipose tissues in mice. We provide evidence for *CLSTN3β* enrichment in multilocular perirenal fat located anatomically in the proximity to both the adrenal gland and sympathetic nerve bundles innervating the kidney in humans. Moreover, transcript levels of *CLSTN3β*, but not *S100b* or *CLSTN3α*, positively correlate with uncoupling protein 1 (*UCP1*) expression in human adipose tissue. Our results further show that *Clsnt3β* is preferentially expressed in brown adipocytes and is highly responsive to changes in environmental temperature and obesity state in mice. Collectively, this brief communication highlights *CLSTN3β* as a hallmark of thermogenic adipose depots in mice and humans.

## Introduction

Cold-induced activation of brown (BAT) and beige adipose tissues enable non-shivering thermogenesis *via* oxidation of substrates (1), a process dependent on the sympathetic innervation of fat. The powerful ability of thermogenic fat to utilize fatty acids and glucose to increase energy expenditure has attracted a growing interest as a therapeutic approach against obesity and related metabolic disorders. Indeed, deep neck BAT volume inversely correlates with body mass index in healthy individuals (2) and its activation *via* short-term cold acclimation increases insulin sensitivity in patients with type II diabetes (3).

Sympathetic innervation of fat by adrenergic neurons is indispensable for norepinephrine (NE)-stimulated lipolysis and activation of uncoupling protein 1 (UCP1) (4, 5), as also demonstrated by chemical or surgical denervation of fat (6, 7). The rich innervation of BAT compared to white adipose tissue (WAT) such as subcutaneous (SAT) or typically white visceral adipose tissue (VAT) is consistent with the high thermogenic capacity of brown adipocytes (8, 9), and elevated innervation is evident in the browning-prone regions of SAT (10). At least three potential molecules have been proposed to exhibit neurotrophic effects in thermogenic fat (11): nerve growth factor (Ngf) (12), neuregulin 4 (Nrg4) (13), and S100b (14). Of interest here, S100b was recently shown to be regulated by a previously unknown adipose-specific calsyntenin 3β (Clstn3β) molecule.

Neuronal calsyntenins (Clstns), members of the cadherin superfamily, are a group of three evolutionarily conserved synapse organizers, originally identified in spinal cord neurons. Expressed throughout the CNS, these type I transmembrane proteins play critical roles in synaptogenesis and synaptic plasticity owing to their cargo-docking function in vesicular transport along axons (15). Clstn3 is mostly expressed in inhibitory neurons and acts as a synaptogenic adhesion molecule *via* formation of functional complexes with Neurexins (Nrx) (16).

The recent discovery of adipose-specific *Clstn3β* by Zeng et al. (2019) (14) surprisingly revealed that BAT expressed a previously unknown form of the *Clstn3* gene and its function facilitates pro-thermogenic innervation *via* chaperone-like activity on the neurotrophic ‘leaderless’ cargo S100b. In contrast to the neuronal Clstn3 (hereafter Clstn3α), Clstn3β is a mammal-specific proetein of the endoplasmic reticulum (ER) membrane and is preferentially expressed in thermogenic adipose tissue in mice (14). *Clstn3β* is a three-exon mRNA, where two of the exons are shared with Clstn3α. The *Clstn3β* protein isoform lacks the *Clstn3α* -specific N-terminal extracellular portion. This unique structural property enables a specific interaction with the neurexins, suggesting that the two proteins have distinct functions. Clstn3β is a novel determinant of the innervation of thermogenic fat, and promotes the ER-localization and secretion of S100b to stimulate neurite outgrowth and thereby potentiates energy expenditure.

While proof-of-concept data supporting the role of Clstn3β in neuritogenesis were elegantly shown using a variety of genetic and chemical tools *in vivo*, the relevance of Clstn3β to human brown/beige fat biology remains unexplored. Basal genetic expression of *Clstn3β* in different cellular fractions of adipose tissue is unknown, and its regulation in response to physiological challenges such as acclimation to cold, thermoneutrality or in the context of obesity is also largely missing. Finally, due to shared sequence homology between the novel *Clstn3β* and the previously annotated neuronal *Clstn3α*, *Clstn3* transcripts have been picked up and annotated in various transcriptomic data sets of mouse (17, 18) and human brown fat (19), therefore the expression of different transcript variants in adipose tissue needs to be clarified.

We therefore aimed to assess adipocyte-specificity and regulation of both *Clstn3α* and *Clstn3β* in various fat depots from humans and mice upon thermogenic activation and deactivation induced by changes in environmental temperature or nutritional stress. We provide evidence for clear enrichment of *CLSTN3β*, but not *CLSTN3α* or *S100b*, in human multilocular BAT and a positive correlation with *UCP1* transcripts across brown (perirenal) and white (subcutaneous) human fat depots. Our mouse data further indicate that *Clstn3β*, but not *Clstn3α*, is an adipocyte-enriched molecule, whose expression is dynamically regulated by metabolic stress.

## Methods

### Human Biopsies

Human perirenal BAT and subcutaneous WAT biopsies were derived from a previously published cohort of healthy kidney donors and collected during nephrectomy (19). The Scientific Ethics Committee of the Capital Region of Copenhagen approved the study protocol journal number H-1-2013-144 and the study was performed in accordance with the Helsinki declaration. Briefly, perirenal fat samples were previously characterized in detail based on transcriptomic and histological profiling and two distinct subtypes of human perirenal BAT was identified; Unilocular and Multilocular. Multilocular perirenal BAT was among other things characterized by increased mitochondrial and thermogenic gene expression and increased tyrosine hydroxylase protein levels. Since these adipose subtypes were distinct across subjects and perirenal regions, the focus of the current study is based on this morphological distinction. In the current study a subset of samples from 16 patients (9 women and 7 men, age 37–68 years, and BMI 16- 37), these consisted of 23 perirenal BAT samples, which were stratified into 12 unilocular and 11 multilocular phenotypes based on histological assessment and subcutaneous fat (abdominal incision, n = 12) were used as white adipose controls. Two subcutaneous, one multilocular perirenal and one unilocular perirenal sample were excluded based on no expression of the reference gene.

### Animals

Mice were tested in accordance with the Danish Animal Experiments Inspectorate (2015-15-0201-00728). Ten-week old C57Bl/6 male mice were used for temperature studies (n = 3 per group) and fat fractionation (n = 12); six-week old male mice were used for the high-fat diet (HFD) study (n = 12). Ob/ob mice were purchased from Jackson laboratories (Bar Harbor, ME, USA, Cat.000632). All mice were kept on a 12-hour light/dark cycle (lights on at 6:00 and off at 18:00) and given *ad libitum* access to chow (Altromin, 1310), unless specified otherwise, and water.

### Exposure to Cold or Thermoneutrality

For cold exposure studies, all mice were first acclimated to 29°C in temperature-controlled incubators (Memmert HPP750Life) for 21 days and then transitioned to 5°C for either 3, 7 or 21 days.

To deactivate thermogenic fat, an independent cohort of mice was pre-exposed to cold (5°C) for 21 days before transition to thermoneutrality (29°C) for either 3 days or 7 days.

### High-Fat Diet Feeding

Mice were given either control diet (D12450B, Research Diets) or high-fat diet (D12492) for 8 weeks.

### Fat Digestion and Isolation of Cell Fractions

Intrascapular BAT was excised from mice and 2 depots were merged per sample (final n = 6). Tissues were washed (10 mM CaCl_2_, 0.5% BSA in PBS) and minced, then collagenized in digestion buffer (2 mg/ml Collagenase type II; Clostridium histolyticum, Sigma Aldrich, Cat.C6885 in PBS containing 10 mM CaCl_2_ and 0.5%-BSA) for 30 min at 37°C. Floating adipocytes (AD) were separated from the pellets (stromal vascular fraction, SVF) by centrifugation (800 rcf, 15 min). The SVF and AD were resuspended and lysed in TRIzol (ThermoFisher Scientific, Cat.15596026).

### Gene Expression Analysis

RNA was isolated using a combined TRIzol-CHCl_3_ and RNeasy spin column (Qiagen, Cat.74106) protocol. cDNA was synthesized from 100 (human samples) 250 ng (mouse samples) of RNA using iScript kit (BioRad, Cat.15596026) and real-time PCR was performed using the Brilliant III Ultrafast SYBR Green QPCR Master Mix (AH Diagnostic, Cat.600883) in CFX384 Bio-Rad System. Mouse raw C_T_ data were normalized to mouse *Ppia*, human data were normalized to *PPIA* following the ΔΔ-C_T_ calculation.

Mouse primers:

Clstn3α, F: GGACAAGGCAACGGGTGAA, R: GCCACAGTCATAAGCCTGAATG
Clstn3β, F: AGGATAACCATAAGCACCAG, R: CTCCGCAGGAACAGCAGCCC
S100b, F: TGGTTGCCCTCATTGATGTCT, R: CCCATCCCCATCTTCGTCC
Ucp1, F: CTGCCAGGACAGTACCCAAG, R: TCAGCTGTTCAAAGCACACA
Adipoq,F: GATGGCACTCCTGGAGAGAA, R: TCTCCAGGCTCTCCTTTCCT
Ppia, F: CATACAGGTCCTGGCATCTTGTC, R: AGACCACATGCTTGCCATCCAG

Human primers:

CLSTN3α, F: ATGACTGTGGGAAGAAGCGG, R: GGCGGATACCAGGGAACAAA
CLSTN3β, F: GCCATCAGCTCTAAGGTCCG, R: CCACAATGATGAGGGTTGCG
S100b, F: TGGCCCTCATCGACGTTTTC, R: ATGTTCAAAGAACTCGTGGCA
UCP1, F: ACCGCAGGGAAAGAAACAGC, R: TCAGATTGGGAGTAGTCCCT
PPIA, F: ACGCCACCGCCGAGAAAAC, R: TGCAAACAGCTCAAAGGAGACGC

### Statistical Analysis

Delta-C_T_ values were used for statistical analysis using GraphPad Prism V.8. For mouse data two-way ANOVA was used for two-variable data sets, otherwise one-way-ANOVA followed by Dunnett post-hoc test was used. Human data were analyzed using one-way-ANOVA (non-parametric, due to lack of normal distribution) with Kruskal Wallis test or correlational analysis with linear regression. Probability levels of 0.05 were considered significant.

## Results

**Figure 1:**
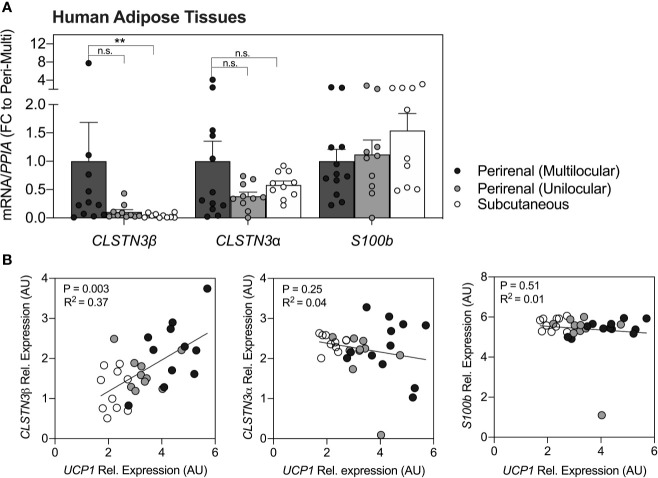
*CLSTN3β* is enriched in human multilocular brown adipose tissue and correlates with *UCP1* expression across fat depots. **(A)** Relative mRNA expression of *CLSTN3β*, *CLSTN3α* and *S100b* in human multilocular or unilocular perirenal brown fat and subcutaneous white fat. Data represent (fold change) means ± SEM. **(B)** Correlational analysis of all three genes vs. *UCP1* across human fat depots (logarithmic values are plotted). Statistical differences are based on one-way-ANOVA with Kruskal Wallis post-test **(A)** or linear regression **(B)**. Asterisks: **p < 0.01.

Quantification of *CLSTN3β* transcripts in human adipose tissues (**Figure 1A**) revealed a significant enrichment in multilocular brown perirenal fat vs. subcutaneous white fat (p < 0.01). No statistical differences were found between multilocular and unilocular perirenal fat for *CLSTN3β*mRNA. Notably, *CLSTN3α* and *S100b* were not enriched in any particular fat depot (p > 0.05). Correlational analysis further revealed that only *CLSTN3β* showed positive relation with *UCP1* expression in all adipose tissue depots examined (p < 0.01, R^2^ = 0.37; **Figure 1B**).

**Figure 2:**
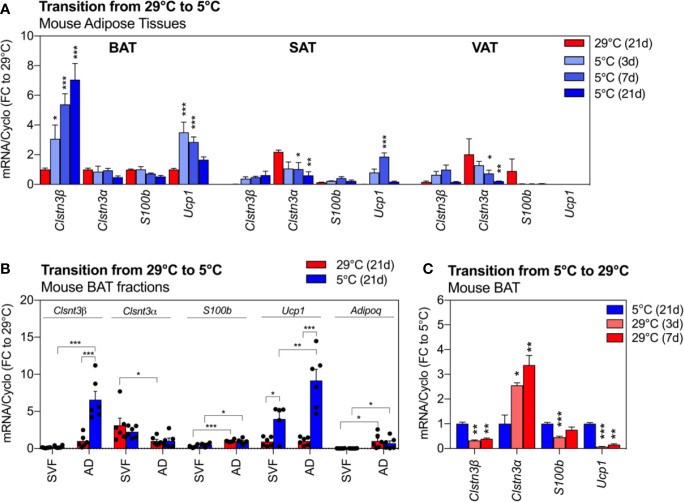
*Clsnt3β* is enriched in mouse brown fat and dynamically regulated by temperature in thermogenic adipose depots. **(A)** Relative gene expression of *Clstn3β*, *Clsnt3α*, *S100b* and *Ucp1* in mouse BAT, SAT and VAT after chronic exposure to thermoneutrality (29°C, 21 days) and transition to cold (5°C) for 3, 7 or 21 days. Data are expressed relative to BAT at 29°C for each gene. **(B)** Brown fat fraction enrichment analysis showing gene expression in BAT stromal vascular fraction (SVF) or adipocytes (AD) of mice exposed chronically to thermoneutrality or cold (21 days). Data are expressed relative to adipocyte fraction at 29°C for each gene. **(C)** Regulation of gene expression in mouse BAT after chronic exposure to cold (5°C, 21 days) and transition to thermoneutrality (29°C) for 3 or 7 days. Data represent means ± SEM and are based on n = 3–6 mice per condition. Overall effects of temperature and depot are reported in graphs (two-way-ANOVA in **A**, **B**; one-way-ANOVA in **C**) and post-hoc comparisons between groups are indicated by asterisks. Asterisks: *p < 0.05, **p < 0.01, ***p < 0.001.

We next assessed the expression of Clstn3*β*, *Clstn3α* and *S100b* across three anatomically distinct murine fat depots and their response to cold exposure (**Figure 2A**). We observed a pronounced enrichment of *Clstn3β* transcripts in BAT compared to WAT depots (p < 0.001), where *Clstn3β* expression was 90% lower in SAT and VAT at 29°C. Exposure to cold further increased the expression of *Clstn3β* in BAT by 7-fold in a time-dependent manner (p < 0.001). This upregulation occurred as early as 3 days of cold exposure, continued to rise and peaked at 21 days post exposure start. Similarly, a trend for cold-induced *Clstn3β* upregulation was observed in WAT from the same mice. The expression of *Ucp1* increased with cold and peaked at 3 days in 5°C (**Figure 2A**). In stark contrast to *Clstn3β*, *Clstn3α* was enriched in white depots (SAT and VAT) and its expression decreased in response to cold (depot: p < 0.0001; temp: p < 0.001). Interestingly, the neurotrophic partner of Clstn3β, *S100b* was not enriched in any particular fat depot but its expression was altered by cold in a depot-specific manner (interaction: p < 0.001). We further demonstrated that *Clstn3β* and *S100b* transcripts were elevated in mature adipocytes from BAT compared to stromal vascular fraction (SVF) (p < 0.001, **Figure 2B**), similarly to *Adiponectin* (p < 0.01) and *Ucp1* (p < 0.05), but not *Clstn3α*, which was most abundant in the stromal-vascular cells (p < 0.01). Furthermore, our data indicate that the increased expression of *Clstn3β* in BAT following cold exposure is primarily due to increased expression of Clstn3β in mature adipocytes.

To further examine the dynamic regulation of *Clstn3β* by changes in environmental temperature, we assessed BAT deactivation in an independent cohort of mice (**Figure 2C**). Similar to *Ucp1*, whose expression drastically decreased after 3 or 7 days at 29°C (−90% to −80% compared to 5°C, p < 0.001 and p < 0.01, respectively), the high expression of *Clstn3β* in BAT was lowered by 60% at 29°C (p values < 0.01). Notably, the expression of *Clstn3α* followed an opposite pattern, with increased transcript levels in BAT of mice exposed to 29°C. *S100b* transcripts were reduced by 30% to 50% with transition to 29°C.

**Figure 3:**
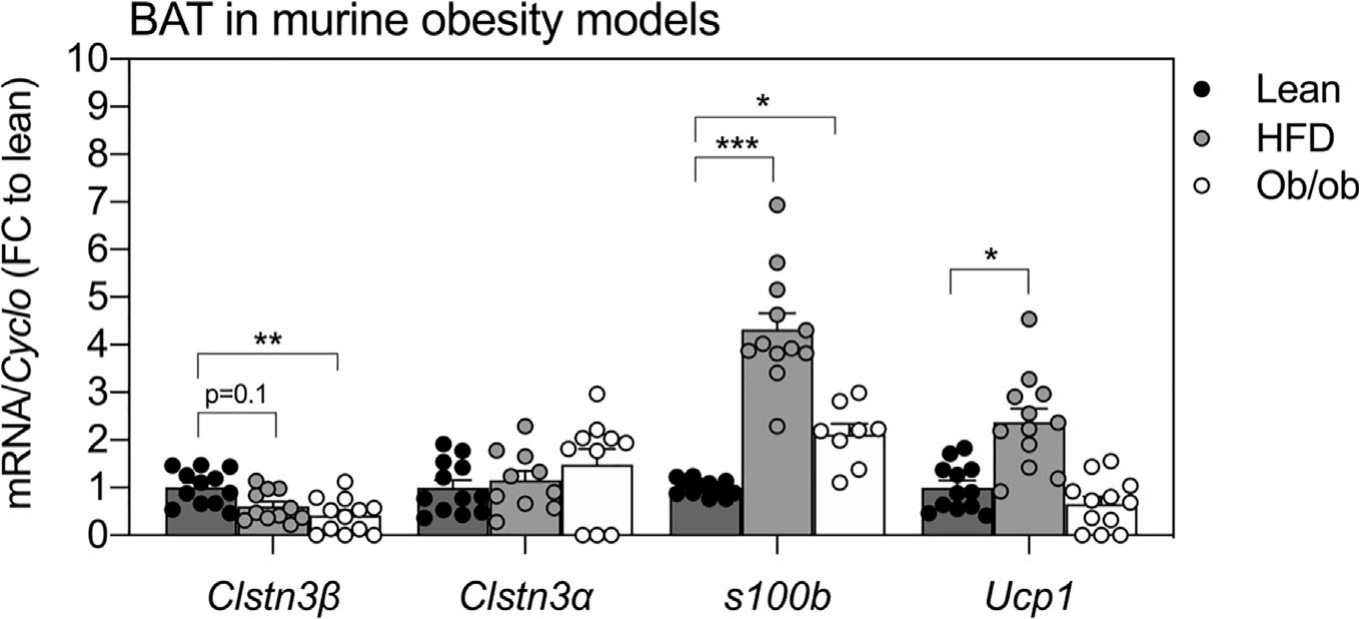
Expression patterns of *Clstn3β* in mouse models of obesity. Gene expression of *Clstn3β*, *Clsnt3α*, *S100b* and *Ucp1* in brown adipose tissue from lean, high-fat diet (HFD) fed or ob/ob mice (n = 11–12 mice per condition). Data represent means ± SEM, expressed relative to lean controls. One-way-ANOVA and post-hoc comparisons between groups are indicated in graphs. Asterisks: *p < 0.05, **p < 0.01, ***p < 0.001.

The expression of *Clstn3β*, but not *Clstn3α*, was also altered in BAT of two standard mouse models of obesity (**Figure 3**). BAT from ob/ob mice showed a 50% reduction in *Clstn3β* transcripts compared to lean mice (p < 0.01) and similar trends were observed in BAT from HFD-fed mice (p = 0.1). In contrast, the expression of *Clstn3α* in BAT was not affected by HFD or genetic leptin ablation. Interestingly, *S100b* was elevated in BAT of HFD-fed mice (p < 0.001) and ob/ob mice (p < 0.05) at this stage.

## Discussion

Rapidly growing pre-clinical and clinical evidence suggests that adrenergic activation of thermogenic fat holds a therapeutic potential to improve metabolic health. The innervation of brown/beige adipose tissue was recently proposed to be governed by the neurotrophic and pro-thermogenic Clstn3β -S100b axis in mice (14). However, the relevance of this novel molecular axis in human adipose tissue, particularly in terms of depot- or phenotype-associated enrichments, is still lacking. Insights into the basic biology of *Clstn3β* and *S100b* in mice, such as cellular distribution and transcript regulation with metabolic stress, are also unexplored. We therefore aimed to provide a simple comprehensive overview of *CLSTN3β*, *CLSTN3α and S100b* transcript enrichments in human and mouse white and beige/brown adipose tissues and their regulation with metabolic stress.

First, we report that *CLSTN3β* is expressed in human perirenal BAT and is enriched in thermogenic perirenal fat (UCP1-positive) compared to the abdomen-derived subcutaneous WAT (UCP1-negative). The elevated *CLSTN3β* in the multilocular perirenal fat, which was previously demonstrated to have increased tyrosine hydroxylase (TH) protein expression (19), suggests a direct correlation of CLSTN3β with adrenergic input, and poses a potential explanation for the multilocular and thermogenically capable phenotype of these adipocytes. Indeed, previous data suggest that BAT from the upper kidney pole and hilus express high levels of β3AR, a pre-thermogenic receptor of adrenergic signaling, as well as PRDM16 (19), a transcriptional regulator of beige adipocyte commitment (20, 21). In addition, the notion that the Clstn3β-S100b complex plays a crucial role in establishing connections between adipose and neuronal cells is further supported by our data illustrating the preferential enrichment of both *Clstn3β* and *S100b* in mature adipocytes.

Our data not only support *Clstn3β* as a BAT-enriched transcript in mice, but further indicate the dynamic regulation of this gene by temperature in thermogenic fat depots. Accordingly, mice exposed to cold showed a drastic and continuous increase in BAT *Clstn3β*, which followed the elevation in *Ucp1*, but contrasted with both *Clstn-3α* and *S100b*. We further found that the high expression of *Clstn3β* in BAT and SAT of cold-acclimated mice is downregulated at 29°C, suggesting that *Clstn-3β* expression highly depends on the energetic demand associated with acclimation to different environmental temperatures. Notably, low levels of *Clstn3α* transcripts were detected in adipose depots. However, the opposite regulation pattern of Clstn3α compared to Clstn3*β*, and its enrichment in the stromal vascular fraction of BAT, do not support a role for this isoform as a neurotrophic factor in adipocytes. Finally, because obesity is associated with ‘whitening’ and dysfunction of BAT (22), the downregulation of *Clstn3β* in BAT from two murine models of obesity suggest *Clstn3β* as a potential marker of BAT alterations. The more pronounced downregulation of *Clstn3β* in BAT of ob/ob mice compared to HFD-fed mice can likely be attributed to the heavier body weight of these mice compared to HFD-fed mice at the same age. In addition, a direct regulation of *Clstn3β* by leptin signaling cannot be excluded. Similar to cold adaptation, the expression of *Clstn3α* was not affected by obesity in BAT, indicating that the β, but not the α variant of the *Clstn3* gene, dynamically responds to pathological events induced by obesity in murine brown fat. The contrasting expression patterns of *Clsnt3β* and *S100b* in BAT in the context of obesity is particularly intriguing. Indeed, an opposite trend was observed for *S100b* (induced by obesity in mouse BAT), and for *Ucp1*, at this stage upon HFD feeding. The initial increase in *Ucp1* transcripts in response to HFD feeding in brown fat in rodents is followed by a decline at longer time points (23), thus raising a tempting speculation that the downregulation of *Clstn3β* in BAT of HFD-fed animals occurs prior to obesity-induced decline in *Ucp1* transcripts. While our findings highlight an interesting regulation of *Clstn3β* in conditions associated with BAT dysfunction, this calls for further investigation about the complex interplay between Clstn3β, S100b and Ucp1 in BAT in the context of obesity. In future studies, it would be informative to assess the expression of *Clstn3β*, *S100b* and *Ucp1* in obese mice across a timeline of brown fat pathology induced by HFD or leptin deficiency. Similarly, given the whitening of BAT (22, 24), reduced thermogenic signature (25) and BAT glucose uptake in humans with obesity (26), the clinical relevance of CLSTN3β in thermogenic fat of obese individuals remains to be determined.

Generally, *Clstn3β* appears as the highly transcriptionally-regulated component of the *Clstn3β* -S100b complex, underlining its possibly initiating role in the formation of new adipo-neuronal connections, thereby potentiating whole-body energy metabolism. Another possibility is that *Clstn3β* is highly regulated by NE signaling as suggested by its enrichment in *UCP1*-positive human brown fat, its responsiveness to environmental temperature, and downregulation in obese state (a condition associated with decreased NE sensitivity), and could act in a positive feedback loop to maintain neuronal connections with thermogenic adipocytes. Interestingly, animal data show that forced Prdm16 expression leads to increased adipose innervation (20), while ablation of this factor in adipocytes has the opposite effect (10). This suggests that certain adipocyte-derived factors, under the control of Prdm16, affect the level of fat innervation. While positive correlations were found between the expression of S100b and Prdm16 in mouse adipose tissue, Clstn3β abundance did not appear to be controlled by Prdm16 (14). Thus, the signaling events and transcriptional complexes controlling *Clstn3β* transcripts in mouse and human currently remain unknown. Finally, it remains to be investigated if *Clstn3*β controls a wider spectrum of BAT-enriched unconventionally secreted molecules that may affect energy homeostasis *via* distal signaling, in addition to controlling locally acting factors such as S100b. Given the underappreciated and poorly understood function of leaderless peptides in metabolic homeostasis, *Clstn3*β may be a good starting point as a potential nodule responsible for intracellular cargo docking and protein shuffling in BAT, similarly to its CNS-enriched counterpart Clstn3α.

Collectively, our data show a clear enrichment pattern and regulation of *Clstn3β* in mouse adipose tissue depots, and reveal *Clstn3β* as a marker of thermogenic adipocytes in humans. Whether CLSTN3β mediates a similar adipose-to-neuron communication in humans remains to be investigated, and could offer a potential strategy to increase energy expenditure in individuals with obesity and diabetes mellitus.

## Data Availability Statement

The original contributions presented in the study are included in the article/supplementary material; further inquiries can be directed to the corresponding author.

## Ethics Statement

The studies involving human participants were reviewed and approved by: The Scientific Ethics Committee of the Capital Region of Copenhagen. The patients/participants provided their written informed consent to participate in this study. The animal study was reviewed and approved by Danish Animal Experiments Inspectorate.

## Author Contributions

BE, CS, and SN supervised the study. KP, NJ, BE, CS, and SN performed hypothesis generation, conceptual design, and data analysis. KP, EB, PP, and KR conducted experiments and data analysis. KP drafted the manuscript. All authors contributed to the article and approved the submitted version.

## Funding

KP and NJ were funded by independent fellowships from Danish Diabetes Academy (DDA) supported by the Novo Nordisk Foundation grant number NNF17SA0031406 (Grant IDs: PD004-18 and PD007-18). The research was supported by internal funding from the Novo Nordisk Foundation Center for Basic Metabolic Research (CBMR) partially funded by an unrestricted donation from the Novo Nordisk Foundation (NNF18CC0034900). The Centre for Physical Activity Research (CFAS) is supported by TrygFonden (grants ID 101390 and ID 20045). During the study period, the Centre of Inflammation and Metabolism (CIM) was supported by a grant from the Danish National Research Foundation (DNRF55).

## Conflict of Interest

The authors declare that the research was conducted in the absence of any commercial or financial relationships that could be construed as a potential conflict of interest.
